# Correction: Preserved endothelial function in young adults with type 1 diabetes

**DOI:** 10.1371/journal.pone.0208865

**Published:** 2018-12-05

**Authors:** The PLOS ONE Staff

## Notice of republication

This article was republished on November 21, 2018, to correct an error introduced during the typesetting process in which a portion of the Results section was included in the Table 1 caption. The publisher apologizes for the error. Please download this article again to view the correct version. The originally published, uncorrected article and the republished, corrected article are provided here for reference.

## Supporting information

S1 File Originally published, uncorrected article. (PDF)

S2 File Republished, corrected article. (PDF)
