# Supplementary material for: Correction: Preserved endothelial function in young adults with type 1 diabetes
Source: PLoS One. 2018 Dec 5;13(12):e0208865. doi: 10.1371/journal.pone.0208865 (PMC6281262; doi:10.1371/journal.pone.0208865)
Supplement: S1 File — (PDF) [file pone.0208865.s001.pdf]

PLOS ONE

RESEARCH ARTICLE

Preserved endothelial function in young adults with type 1 diabetes

Martin Heier[1,2,3]*, Cathrine Nygaard Espeland[4], Cathrine Brunborg[5], Ingebjørg Seljeflot[2,6], Hanna Dis Margeirsdottir[2,3,7], Kristian F. Hanssen[2,3,8], Drude Fugelseth[2,4], Knut Dahl-Jørgensen[1,2,3]

1 Pediatric Department, Oslo University Hospital, Oslo, Norway, **2** Institute of Clinical Medicine, Faculty of Medicine, University of Oslo, Oslo, Norway, **3** Oslo Diabetes Research Centre, Oslo, Norway, **4** Department of Neonatal Intensive Care, Oslo University Hospital, Oslo, Norway, **5** Oslo Centre for Biostatistics and Epidemiology, Research Support Services, Oslo University Hospital, Oslo, Norway, **6** Center for Clinical Heart Research and Department of Cardiology, Oslo University Hospital, Oslo, Norway, **7** Pediatric Department, Akershus University Hospital, Lørenskog, Norway, **8** Department of Endocrinology, Oslo University Hospital, Oslo, Norway

* martin.heier@medisin.uio.no

Check for updates

OPEN ACCESS

Citation: Heier M, Espeland CN, Brunborg C, Seljeflot I, Margeirsdottir HD, Hanssen KF, et al. (2018) Preserved endothelial function in young adults with type 1 diabetes. PLoS ONE 13(10): e0206523. https://doi.org/10.1371/journal.pone.0206523

Editor: Petter Bjornstad, University of Colorado Denver School of Medicine, UNITED STATES

Received: July 18, 2018

Accepted: October 15, 2018

Published: October 25, 2018

Copyright: © 2018 Heier et al. This is an open access article distributed under the terms of the Creative Commons Attribution License, which permits unrestricted use, distribution, and reproduction in any medium, provided the original author and source are credited.

Data Availability Statement: All relevant data are within the paper and its Supporting Information files.

Funding: The Norwegian Extra Foundation for Health and Rehabilitation funded HDM to initiate the "Atherosclerosis and Childhood Diabetes" study. https://www.extrastiftelsen.no The funders had no role in study design, data collection and analysis, decision to publish, or preparation of the manuscript.

Abstract

Background and aim

Endothelial dysfunction is involved in the pathogenesis of atherosclerosis and is typically present in older adults with type 1 diabetes (T1D). In young adults, we aimed to assess the impact of T1D on endothelial function as detected by digital peripheral arterial tonometry (PAT) and its relationship with cardiovascular risk factors and long term glycemic control.

Materials and methods

Reactive hyperemia index (RHI) as a measure of endothelial function was assessed by PAT in 46 T1D patients and 32 healthy controls. All were participants in the "Atherosclerosis and Childhood Diabetes" study, with baseline values registered five years previously. Annual measurements of HbA1c for assessment of glycemic burden were provided by the Norwegian Childhood Diabetes Registry.

Results

The diabetes patients had a mean age of 20.8 years, a median duration of diabetes of 10.0 years and a mean HbA1c of 8.7%. RHI was not significantly decreased in the diabetes group, mean 2.00 (SD = 0.59) vs. 2.21 (SD = 0.56), p = .116. There was no gender difference or any associations with traditional risk factors. Furthermore, there was no significant association between RHI and either HbA1c or long term glycemic burden.

Conclusions

RHI as a measure of endothelial function was preserved in young adults with T1D compared with healthy controls.

Competing interests: The authors have declared that no competing interests exist.

Introduction

Endothelial dysfunction is a frequent characteristic of early atherogenesis.[1, 2] Flow-mediated dilatation (FMD) has been the most widely used non-invasive method of assessing endothelial function. It reflects the bioavailability of endothelial-derived nitric oxide (NO),[3] and impaired FMD is associated with cardiovascular disease (CVD) events.[4, 5] FMD is, however, operator dependent and requires strict technical and methodological standardization.[6] Perhaps overcoming these limitations is a device that applies peripheral arterial tonometry (PAT), EndoPAT (Itamar Medical Ltd, Caesarea, Israel). EndoPAT requires less training and is largely automated. Sensors placed on each index finger measure changes in blood volume in the vasculature before and after a 5-minute occlusion of the brachial artery in the non-dominant arm. The contralateral arm serves as a control, facilitating adjustment for non-endothelial dependent factors. The adjusted post-occlusion to pre-occlusion ratio, the reactive hyperemia index (RHI), is calculated. RHI has been shown to correlate well with invasive measurement of coronary endothelial function,[7] predict adverse cardiovascular events in a 7-year follow-up study [8] and was associated with conventional risk factors in a community-based cohort of adults.[9] As with FMD, changes in RHI are also largely mediated by NO.[10] Despite this, a majority of studies show a poor correlation between FMD and RHI, suggesting that they provide different and unique information about vasoreactivity.[11–16]

Patients with type 1 diabetes (T1D) are prone to accelerated atherosclerosis, leading to increased morbidity and mortality from CVD.[17, 18] There is, however, insufficient knowledge about the early phases of atherosclerosis, partially due to the lack of clinically useful non-invasive techniques to precisely assess the subclinical stage. Endothelial function is frequently impaired in adult patients with T1D.[19] In children and adolescents with T1D, most, but not all studies of FMD have demonstrated reduced endothelial function in patients with T1D compared with healthy control subjects.[20–24] Studies applying RHI in this age group have also largely shown reduced values in patients with T1D.[25–27] None of these studies were longitudinal.

We hypothesized that T1D would negatively affect endothelial function. We aimed to test our hypothesis by assessing the impact of T1D on endothelial function as detected by PAT and its relationship with cardiovascular risk factors and long term glycemic control.

Materials and methods

Study population

The baseline examinations in the population-based "Atherosclerosis and Childhood Diabetes" study were performed from 2006–2008.[28] As part of the 5-year follow-up, 2011–2013, participants above 18 years of age were invited to take part in the present sub-study. Among the healthy control subjects, 17 had baseline values and 15 were recruited in the 5-year follow-up. In order to isolate the effect of T1D in early atherosclerosis, exclusion criteria were smoking, pregnancy, current infectious disease, hypertension (above 90th percentile), chronic diseases other than diabetes or any kind of long term medication. The diabetes patients all received intensified insulin injection treatment (> 4 daily injections) or used insulin pumps from the time of diagnosis. None had overt retinopathy or nephropathy. A total of 46 diabetes patients and 32 controls were included. They all gave their written informed consent. The protocol was approved by the Norwegian Regional Committee for Research Ethics, and the study was conducted according to the Declaration of Helsinki.

Laboratory analyses

All examinations were performed after an overnight fast. The baseline clinical examination has been described previously,[28] and baseline data was available for all diabetes patients and 17 of the controls.

Annual HbA1c values from 2000 to 2012 were obtained from the Norwegian Childhood Diabetes Registry, and they were all measured at the same DCCT-standardized laboratory using high performance liquid chromatography (Variant; Bio-Rad, Richmond, CA, USA), the inter-assay coefficient of variation (CV) $< 3\%$. Each patient had on average 8 annual HbA1c measurements (range 3–12), and these were used to calculate mean HbA1c at baseline and follow-up. Diabetes duration multiplied by mean HbA1c at each time point provided an estimate of glycemic burden.

Routine laboratory analyses were performed by conventional methods.

Endothelial function

Digital PAT was performed using EndoPAT 2000 (Itamar Medical Ltd, Caesarea, Israel) as part of the 5-year follow-up of the "Atherosclerosis and Childhood Diabetes" study. The patients were examined in the supine position on a bed in a quiet and dimly lit room. Plethysmographic bio-sensors were placed on the index finger of both hands. From these, arterial pulsatile volume changes were recorded. After registering a five minute baseline signal, a brachial blood pressure cuff on the non-dominant upper arm was inflated to approximately 200 mmHg for exactly five minutes. This period of ischemia resulted in vasodilation followed by reactive hyperemia when blood flow returned. The PAT signal was recorded for another five minutes. A post-occlusion to pre-occlusion ratio was calculated by the EndoPAT software, resulting in a reactive hyperemia index. An RHI score of 1.67 and below is considered abnormal, and a score above 2.00 is recommended.[7]

Statistical analysis

Demographic and clinical data are presented as either proportions, means with their standard deviations (SD) or medians with the 25th and 75th percentile. Differences in continuous variables between groups were tested with the Student *t*-test for normally distributed data, alternatively the Mann-Whitney *U*-test for non-normally distributed data. Correlation analyses between continuous variables were performed using Pearson's correlation coefficient (r) or Spearman's rho (ρ) when appropriate. Univariate linear regression analysis was performed to study the association between current conventional risk factors (systolic blood pressure, diastolic blood pressure, total cholesterol, LDL cholesterol, HDL cholesterol, triglycerides, apolipoprotein B, apolipoprotein A-I, HbA1c, waist circumference, body mass index (BMI) and diabetes duration) as exposure variables with RHI as the outcome variable. To identify possible confounders, we studied all variables that could influence the outcome. Only variables with significant relationships with both the exposure and the outcome variables were considered as possible confounders and included in a multivariate analysis. Adjustment for multiple confounding factors was done using multivariate linear regression analysis with a manual backward elimination procedure. A significance level of 5% was used. All statistical analyses were performed using the SPSS software package for Mac, version 19.0 (SPSS, Chicago, IL).

Results

The clinical and metabolic characteristics of the participants are shown in Table 1. The diabetes patients had greater waist circumference compared with the controls, as well as higher

Table 1. Clinical and metabolic characteristics.

	Baseline			5-year follow-up		
	**Diabetes**	**Controls**	**p-value**	**Diabetes**	**Controls**	**p-value**
n	46	17		46	32	
Diabetes duration (years)[1]	5.3 (3.4, 9.6)			10.0 (8.1, 14.3)		
Insulin pump users n (%)	20 (43.5)			23 (50.0)		
Age (years)	16.0 (1.8)	15.4 (2.0)	.286	20.8 (1.8)	21.1 (1.9)	.519
Girls, n (%)	22 (47.8)	9 (52.9)	.718	22 (47.8)	17 (53.1)	.645
Height (cm)	170.4 (8.9)	170.3 (7.8)	.940	174.5 (8.5)	175.3 (9.1)	.688
Weight (kg)	66.5 (15.0)	59.0 (12.2)	.070	78.8 (15.3)	72.6 (15.0)	.080
BMI (kg/m^2)[1]	21.6 (19.6, 25.4)	19.0 (17.9, 23.7)	.017	23.9 (22.6, 28.1)	22.7 (20.5, 26.0)	.037
Waist circumference (cm)	76.5 (9.8)	69.5 (6.4)	.003	84.1 (10.5)	78.0 (10.1)	.012
Systolic blood pressure (mmHg)	105.2 (11.0)	104.6 (10.8)	.866	115.8 (11.5)	114.6 (8.9)	.616
Diastolic blood pressure (mmHg)	62.2 (9.6)	60.5 (6.2)	.520	70.0 (8.9)	69.9 (7.4)	.943
Pulse pressure	43.0 (7.6)	44.1 (9.3)	.631	45.7 (9.2)	44.7 (8.9)	.605
HbA1c (%)	8.2 (1.1)	5.3 (0.3)	< .001	8.7 (1.4)	5.2 (0.3)	< .001
Mean HbA1c (%)	7.9 (1.1)			8.3 (1.0)		
Glycemic burden (% x years)	52.3 (34.9)			94.0 (36.3)		
Total cholesterol (mmol/L)	4.5 (0.7)	4.0 (0.7)	.014	4.8 (1.0)	4.5 (0.9)	.138
HDL cholesterol (mmol/L)	1.7 (0.4)	1.6 (0.4)	.325	1.6 (0.4)	1.6 (0.5)	.914
LDL cholesterol (mmol/L)	2.5 (0.6)	2.1 (0.6)	.042	2.7 (0.8)	2.5 (0.7)	.150
Triglycerides (mmol/L)[1]	0.7 (0.6, 0.8)	0.6 (0.4, 0.7)	.248	1.0 (0.7, 1.4)	1.0 (0.7, 1.5)	.681
Apolipoprotein B (g/L)	0.74 (0.16)	0.60 (0.13)	.002	0.92 (0.23)	0.81 (0.20)	.041
Apolipoprotein A-I (g/L)	1.44 (0.24)	1.18 (0.38)	.021	1.56 (0.32)	1.55 (0.35)	.936
Urine Albumin/Creatinine (mg/mmol)[1]	0.50 (0.30, 1.28)	0.77 (0.35, 1.64)	.669	0.56 (0.24, 1.25)	0.30 (0.14, 0.99)	.405
Reactive Hyperemia Index (RHI)				2.00 (0.59)	2.21 (0.56)	.116

Mean values (SD).

[1] Median (25th and 75th percentile).

RHI tended to be lower in the diabetes group compared with controls, but the difference was not significant, mean 2.00 (SD = 0.59) vs. 2.21 (SD = 0.56), p = .116. We found no significant difference between the genders among all participants (mean girls 2.08 (SD = 0.60) vs. boys 2.09 (SD = 0.57), p = .929), in the diabetes group (mean girls 2.01 (SD = 0.59) vs. boys 1.99 (SD = 0.61), p = .888), or in the control group (mean girls 2.17 (SD = 0.62) vs. boys 2.26 (SD = 0.49), p = .646). There was no significant correlation between RHI and HbA1c, mean HbA1c or glycemic burden, either at baseline or at the 5-year follow-up. RHI was not significantly associated with any current conventional cardiovascular risk factors in either group. Using the cut-off value recommended by the manufacturer of 1.67 to determine endothelial dysfunction, we found no significant difference between the groups, p = .447 (Fig 1).

https://doi.org/10.1371/journal.pone.0206523.t001

HbA1c and apolipoprotein B. At baseline they also had higher BMI, total cholesterol, LDL cholesterol and apolipoprotein A-I. The participants in both groups had otherwise similar characteristics.

Discussion

The main finding in our study was the lack of significant difference in RHI between young adult patients with T1D and healthy control subjects. This is in contrast to previous well conducted studies with similar sample size (44 and 23 patients with T1D, respectively).[25, 26] The participants in these studies, however, were slightly younger than in our study, and all had not completed puberty. Possibly, pubertal stage could explain these differences, as later studies in healthy children have shown that RHI increases during puberty.[29, 30] Difference in pubertal stage might also be the reason Mahmud et al. found lower values in boys with T1D compared with girls.[31] Pareyn et al., however, found lower values in girls with T1D.[27] This

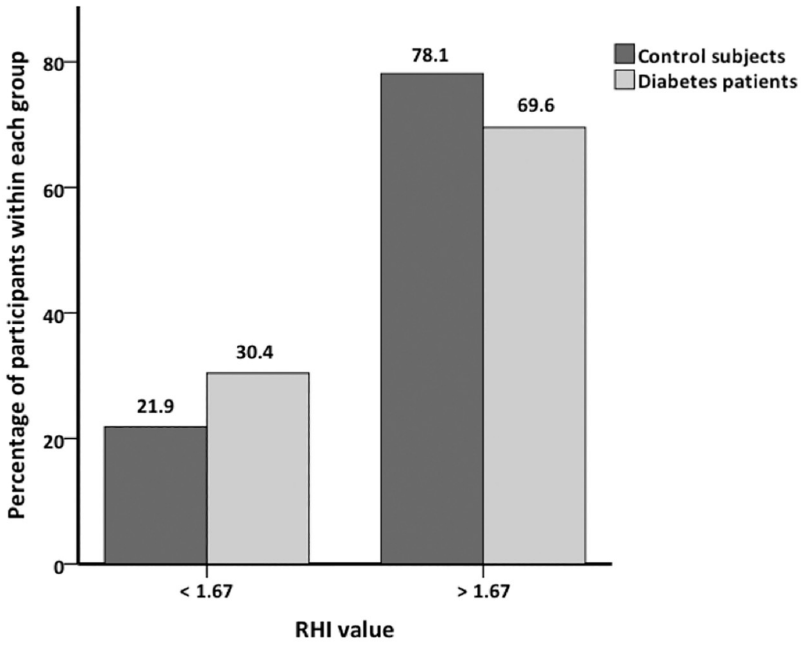

Fig 1. Percentage of participants above and below RHI = 1.67.

https://doi.org/10.1371/journal.pone.0206523.g001

discrepancy may be due to chance, as we were unable to demonstrate any gender difference, in line with a larger study in healthy children.[32]

As Fig 1 illustrates, 30.4% of the diabetes patients were below the cut-off value set by the manufacturer to indicate endothelial dysfunction. This was also the case for 21.9% of the controls, and there was no significant difference between the groups. Scaramuzza et al. have demonstrated considerably higher percentages (76.7% and 81.8%) in adolescents with T1D during one year of follow-up.[33] We find it unlikely that 21.9% of healthy young adults have impaired endothelial function. Thus, the relatively high number of abnormal values among both diabetes patients and control subjects suggests that the recommended cut-off value might not be ideal for cardiovascular risk assessment in this age group.

In a cross-sectional study of middle-aged healthy volunteers, associations between RHI and male gender, BMI, total-/HDL-cholesterol, diabetes, smoking and lipid-lowering therapy were reported.[9] Also, in healthy adolescents RHI was associated with soluble intercellular adhesion molecule-1, insulin resistance and saturated fatty acids.[32] Contrary to this, smaller studies in healthy subjects[15, 30] and in young patients with T1D[25, 27] only showed significant associations with RHI and pubertal stage. In our study, there were no significant associations between traditional cardiovascular risk factors and RHI in either group. These disparities are likely due to differences in sample size.

Previous studies are also inconsistent with regard to the association between RHI and HbA1c.[25–27, 31, 33, 34] The present study provides reliable longitudinal assessments of HbA1c, in many cases annually since diagnosis. The resulting measure of glycemic burden, however, was not associated with RHI.

Inherent strengths of this study include the prospective design and several longitudinal measurements of HbA1c for each patient. Major limitations are the small number of study subjects, particularly controls with baseline values, and the lack of serial measurements of RHI.

In conclusion, we did not find reduced endothelial function, as measured by EndoPAT, in young adults with T1D compared with healthy controls. Furthermore, we did not find any

gender difference or significant associations with traditional cardiovascular risk factors or glycemic burden. These results suggest that EndoPAT has limited value in CVD risk assessment in young adults with T1D, but larger and longitudinal studies are needed.

Supporting information

S1 File. Dataset for endothelial function.
(XLSX)

Acknowledgments

The authors would like to acknowledge Eva B. Lindseth for all her work in recruiting patients, collecting and organizing data. We are also grateful to the Norwegian Childhood Diabetes Registry for providing HbA1c data.

Author Contributions

Conceptualization: Hanna Dis Margeirsdottir, Kristian F. Hanssen, Knut Dahl-Jørgensen.

Formal analysis: Martin Heier, Cathrine Brunborg.

Funding acquisition: Hanna Dis Margeirsdottir, Knut Dahl-Jørgensen.

Investigation: Martin Heier, Cathrine Nygaard Espeland, Hanna Dis Margeirsdottir.

Resources: Ingebjørg Seljeflot, Drude Fugelseth.

Supervision: Kristian F. Hanssen, Knut Dahl-Jørgensen.

Writing – original draft: Martin Heier.

References

1. Celermajer DS, Sorensen KE, Gooch VM, Spiegelhalter DJ, Miller OI, Sullivan ID et al. Non-invasive detection of endothelial dysfunction in children and adults at risk of atherosclerosis. Lancet. 1992; 340: 1111–1115. PMID: 1359209
2. Juonala M, Viikari JS, Laitinen T, Marniemi J, Helenius H, Rönnemaa T et al. Interrelations between brachial endothelial function and carotid intima-media thickness in young adults: the cardiovascular risk in young Finns study. Circulation. 2004; 110: 2918–2923. https://doi.org/10.1161/01.CIR.0000147540.88559.00 PMID: 15505080
3. Mullen MJ, Kharbanda RK, Cross J, Donald AE, Taylor M, Vallance P et al. Heterogenous nature of flow-mediated dilatation in human conduit arteries in vivo: relevance to endothelial dysfunction in hypercholesterolemia. Circ Res. 2001; 88: 145–151. PMID: 11157665
4. Suwaidi JA, Hamasaki S, Higano ST, Nishimura RA, Holmes DR, Lerman A. Long-term follow-up of patients with mild coronary artery disease and endothelial dysfunction. Circulation. 2000; 101: 948–954. PMID: 10704159
5. Yeboah J, Crouse JR, Hsu FC, Burke GL, Herrington DM. Brachial flow-mediated dilation predicts incident cardiovascular events in older adults: the Cardiovascular Health Study. Circulation. 2007; 115: 2390–2397. https://doi.org/10.1161/CIRCULATIONAHA.106.678276 PMID: 17452608
6. Thijssen DH, Black MA, Pyke KE, Padilla J, Atkinson G, Harris RA et al. Assessment of flow-mediated dilation in humans: a methodological and physiological guideline. Am J Physiol Heart Circ Physiol. 2011; 300: H2–12. https://doi.org/10.1152/ajpheart.00471.2010 PMID: 20952670
7. Bonetti PO, Pumper GM, Higano ST, Holmes DRJ, Kuvin JT, Lerman A. Noninvasive identification of patients with early coronary atherosclerosis by assessment of digital reactive hyperemia. J Am Coll Cardiol. 2004; 44: 2137–2141. https://doi.org/10.1016/j.jacc.2004.08.062 PMID: 15582310
8. Rubinshtein R, Kuvin JT, Soffler M, Lennon RJ, Lavi S, Nelson RE et al. Assessment of endothelial function by non-invasive peripheral arterial tonometry predicts late cardiovascular adverse events. Eur Heart J. 2010; 31: 1142–1148. https://doi.org/10.1093/eurheartj/ehq010 PMID: 20181680

9. Hamburg NM, Keyes MJ, Larson MG, Vasan RS, Schnabel R, Pryde MM et al. Cross-sectional relations of digital vascular function to cardiovascular risk factors in the Framingham Heart Study. Circulation. 2008; 117: 2467–2474. https://doi.org/10.1161/CIRCULATIONAHA.107.748574 PMID: 18458169

10. Nohria A, Gerhard-Herman M, Creager MA, Hurley S, Mitra D, Ganz P. Role of nitric oxide in the regulation of digital pulse volume amplitude in humans. J Appl Physiol. 2006; 101: 545–548. https://doi.org/10.1152/japplphysiol.01285.2005 PMID: 16614356

11. Hamburg NM, Palmisano J, Larson MG, Sullivan LM, Lehman BT, Vasan RS et al. Relation of brachial and digital measures of vascular function in the community: the Framingham heart study. Hypertension. 2011; 57: 390–396. https://doi.org/10.1161/HYPERTENSIONAHA.110.160812 PMID: 21263120

12. Schnabel RB, Schulz A, Wild PS, Sinning CR, Wilde S, Eleftheriadis M et al. Noninvasive vascular function measurement in the community: cross-sectional relations and comparison of methods. Circ Cardiovasc Imaging. 2011; 4: 371–380. https://doi.org/10.1161/CIRCIMAGING.110.961557 PMID: 21551420

13. Onkelinx S, Cornelissen V, Goetschalckx K, Thomaes T, Verhamme P, Vanhees L. Reproducibility of different methods to measure the endothelial function. Vasc Med. 2012; 17: 79–84. https://doi.org/10.1177/1358863X12436708 PMID: 22402933

14. Lind L. Relationships between three different tests to evaluate endothelium-dependent vasodilation and cardiovascular risk in a middle-aged sample. J Hypertens. 2013; 31: 1570–1574. https://doi.org/10.1097/HJH.0b013e3283619d50 PMID: 23625114

15. Wilk G, Osmenda G, Matusik P, Nowakowski D, Jasiewicz-Honkisz B, Ignacak A et al. Endothelial function assessment in atherosclerosis: comparison of brachial artery flow-mediated vasodilation and peripheral arterial tonometry. Pol Arch Med Wewn. 2013; 123: 443–452. PMID: 24025637

16. Kuvin JT, Patel AR, Sliney KA, Pandian NG, Sheffy J, Schnall RP et al. Assessment of peripheral vascular endothelial function with finger arterial pulse wave amplitude. Am Heart J. 2003; 146: 168–174. https://doi.org/10.1016/S0002-8703(03)00094-2 PMID: 12851627

17. Laing SP, Swerdlow AJ, Slater SD, Burden AC, Morris A, Waugh NR et al. Mortality from heart disease in a cohort of 23,000 patients with insulin-treated diabetes. Diabetologia. 2003; 46: 760–765. https://doi.org/10.1007/s00125-003-1116-6 PMID: 12774166

18. Krolewski AS, Kosinski EJ, Warram JH, Leland OS, Busick EJ, Asmal AC et al. Magnitude and determinants of coronary artery disease in juvenile-onset, insulin-dependent diabetes mellitus. Am J Cardiol. 1987; 59: 750–755. PMID: 3825934

19. De Vriese AS, Verbeuren TJ, Van de Voorde J, Lameire NH, Vanhoutte PM. Endothelial dysfunction in diabetes. Br J Pharmacol. 2000; 130: 963–974. https://doi.org/10.1038/sj.bjp.0703393 PMID: 10882379

20. Bradley TJ, Slorach C, Mahmud FH, Dunger DB, Deanfield J, Deda L et al. Early changes in cardiovascular structure and function in adolescents with type 1 diabetes. Cardiovasc Diabetol. 2016; 15: 31. https://doi.org/10.1186/s12933-016-0351-3 PMID: 26879273

21. Babar GS, Zidan H, Widlansky ME, Das E, Hoffmann RG, Daoud M et al. Impaired endothelial function in preadolescent children with type 1 diabetes. Diabetes Care. 2011; 34: 681–685. https://doi.org/10.2337/dc10-2134 PMID: 21289230

22. Jarvisalo MJ, Raitakari M, Toikka JO, Putto-Laurila A, Rontu R, Laine S et al. Endothelial dysfunction and increased arterial intima-media thickness in children with type 1 diabetes. Circulation. 2004; 109: 1750–1755. https://doi.org/10.1161/01.CIR.0000124725.46165.2C PMID: 15023875

23. Sochett E, Noone D, Grattan M, Slorach C, Moineddin R, Elia Y et al. Relationship between serum inflammatory markers and vascular function in a cohort of adolescents with type 1 diabetes. Cytokine. 2017; 99: 233–239. https://doi.org/10.1016/j.cyto.2017.07.013 PMID: 28760408

24. Cé GV, Rohde LE, da Silva AM, Puñales MK, Coutinho MK, de Castro AC et al. Endothelial dysfunction is related to poor glycemic control in adolescents with type 1 diabetes under 5 years of disease: evidence of metabolic memory. J Clin Endocrinol Metab. 2011; 96: 1493–1499. https://doi.org/10.1210/jc.2010-2363 PMID: 21346068

25. Haller MJ, Stein J, Shuster J, Theriaque D, Silverstein J, Schatz DA et al. Peripheral artery tonometry demonstrates altered endothelial function in children with type 1 diabetes. Pediatr Diabetes. 2007; 8: 193–198. https://doi.org/10.1111/j.1399-5448.2007.00246.x PMID: 17659060

26. Mahmud FH, Van Uum S, Kanji N, Thiessen-Philbrook H, Clarson CL. Impaired endothelial function in adolescents with type 1 diabetes mellitus. J Pediatr. 2008; 152: 557–562. https://doi.org/10.1016/j.jpeds.2007.08.044 PMID: 18346515

27. Pareyn A, Allegaert K, Asscherickx W, Peirsman E, Verhamme P, Casteels K. Impaired endothelial function in female adolescents with type 1 diabetes measured by peripheral artery tonometry. Eur J Pediatr. 2013; 172: 1017–1022. https://doi.org/10.1007/s00431-013-1988-5 PMID: 23525544

28. Margeirsdottir HD, Stensaeth KH, Larsen JR, Brunborg C, Dahl-Jorgensen K. Early signs of atherosclerosis in diabetic children on intensive insulin treatment: a population-based study. Diabetes Care. 2010; 33: 2043–2048. https://doi.org/10.2337/dc10-0505 PMID: 20530748

29. Bhangoo A, Sinha S, Rosenbaum M, Shelov S, Ten S. Endothelial function as measured by peripheral arterial tonometry increases during pubertal advancement. Horm Res Paediatr. 2011; 76: 226–233. https://doi.org/10.1159/000328455 PMID: 21778688

30. Radtke T, Khattab K, Eser P, Kriemler S, Saner H, Wilhelm M. Puberty and microvascular function in healthy children and adolescents. J Pediatr. 2012; 161: 887–891. https://doi.org/10.1016/j.jpeds.2012.04.033 PMID: 22632874

31. Mahmud FH, Earing MG, Lee RA, Lteif AN, Driscoll DJ, Lerman A. Altered endothelial function in asymptomatic male adolescents with type 1 diabetes. Congenit Heart Dis. 2006; 1: 98–103. https://doi.org/10.1111/j.1747-0803.2006.00015.x PMID: 18377552

32. Chen Y, Osika W, Dangardt F, Gan LM, Strandvik B, Friberg P. High levels of soluble intercellular adhesion molecule-1, insulin resistance and saturated fatty acids are associated with endothelial dysfunction in healthy adolescents. Atherosclerosis. 2010; 211: 638–642. https://doi.org/10.1016/j.atherosclerosis.2010.03.013 PMID: 20362293

33. Scaramuzza AE, Redaelli F, Giani E, Macedoni M, Giudici V, Gazzarri A et al. Adolescents and young adults with type 1 diabetes display a high prevalence of endothelial dysfunction. Acta Paediatr. 2015; 104: 192–197. https://doi.org/10.1111/apa.12877 PMID: 25424745

34. Shachor-Meyouhas Y, Pillar G, Shehadeh N. Uncontrolled type 1 diabetes mellitus and endothelial dysfunction in adolescents. Isr Med Assoc J. 2007; 9: 637–640. PMID: 17939622
